# Imbalances in the Development of Muscle and Tendon as Risk Factor for Tendinopathies in Youth Athletes: A Review of Current Evidence and Concepts of Prevention

**DOI:** 10.3389/fphys.2017.00987

**Published:** 2017-12-01

**Authors:** Falk Mersmann, Sebastian Bohm, Adamantios Arampatzis

**Affiliations:** ^1^Department of Training and Movement Sciences, Humboldt-Universität zu Berlin, Berlin, Germany; ^2^Berlin School of Movement Science, Berlin, Germany

**Keywords:** muscle, tendon, adaptation, athletes, adolescence, tendinopathy, imbalance

## Abstract

Tendons feature the crucial role to transmit the forces exerted by the muscles to the skeleton. Thus, an increase of the force generating capacity of a muscle needs to go in line with a corresponding modulation of the mechanical properties of the associated tendon to avoid potential harm to the integrity of the tendinous tissue. However, as summarized in the present narrative review, muscle and tendon differ with regard to both the time course of adaptation to mechanical loading as well as the responsiveness to certain types of mechanical stimulation. Plyometric loading, for example, seems to be a more potent stimulus for muscle compared to tendon adaptation. In growing athletes, the increased levels of circulating sex hormones might additionally augment an imbalanced development of muscle strength and tendon mechanical properties, which could potentially relate to the increasing incidence of tendon overload injuries that has been indicated for adolescence. In fact, increased tendon stress and strain due to a non-uniform musculotendinous development has been observed recently in adolescent volleyball athletes, a high-risk group for tendinopathy. These findings highlight the importance to deepen the current understanding of the interaction of loading and maturation and demonstrate the need for the development of preventive strategies. Therefore, this review concludes with an evidence-based concept for a specific loading program for increasing tendon stiffness, which could be implemented in the training regimen of young athletes at risk for tendinopathy. This program incorporates five sets of four contractions with an intensity of 85–90% of the isometric voluntary maximum and a movement/contraction duration that provides 3 s of high magnitude tendon strain.

## Introduction

Tendinopathy is a clinical condition that is associated with pathological processes within the tendon and pain (Fredberg and Stengaard-Pedersen, 2008). In specific sport disciplines (i.e., jump disciplines) about every second athlete develops a tendinopathy during the athletic career and most individuals suffer from chronic symptoms (Lian et al., 2005). Until recently, only few information was available on the prevalence of tendinopathy in children and adolescents. However, the available literature indicates that tendon overload injury is a common issue in youth sports and that the prevalence increases during maturation (Simpson et al., 2016). Some reports indicate that tendinopathy is the most frequent overuse injury in adolescent athletes (Le Gall et al., 2006). The present narrative review explores the hypothesis that an imbalanced adaptation of muscle and tendon might contribute to the etiology of tendinopathies in youth sports.

In the production of movement, muscles and tendons work as a unit, in which the tendon transmits the forces generated by the muscle to the skeleton (Józsa and Kannus, 1997; Nigg and Herzog, 2007). The resultant stress that is applied to the tendon (i.e., force normalized to tendon cross-sectional area) is a measure of absolute load on the tissue irrespective of its dimensions. Yet, ultimate stress (i.e., stress at tendon failure) varies markedly across different tendons and species (LaCroix et al., 2013). In contrast, the ultimate strain of tendons is remarkably constant (Abrahams, 1967; Loitz et al., 1989; LaCroix et al., 2013; Shepherd and Screen, 2013). This means that, from a mechanobiological perspective, tendon strain is the most adequate indicator of the mechanical demand for the tendon as a result of loading. Though it has been argued that an increase of stress might still contribute to either physiological adaptation (Wiesinger et al., 2015) or pathological changes (Couppé et al., 2013), experimental evidence clearly highlights habitual tendon strain as the most crucial parameter for the risk of injury (Wren et al., 2003; LaCroix et al., 2013; Veres et al., 2013). Therefore, an increase of the strength-generating capacity of a muscle needs to go in line with a corresponding modulation of the mechanical properties of the respective tendon. The increase of tendon stiffness (i.e., the slope of the force-elongation relationship) serves as a protective mechanism for integrity of the tendinous tissue and has been frequently observed in humans to accompany strength gains due to both mechanical loading (Kubo et al., 2001a; Arampatzis et al., 2007; Kongsgaard et al., 2007) as well as maturation (Kubo et al., 2001b, 2014; O'Brien et al., 2010b; Waugh et al., 2012; Mersmann et al., 2016). Yet, there is now growing evidence that the adaptation of muscle and tendon does not necessarily proceed in a uniform manner during a training process. In young athletes, maturation acts as an additional stimulus on the development of the muscle-tendon unit, which could potentially further challenge the uniformity of muscle strength and tendon stiffness changes. In the following chapters, we will summarize potentially influential factors for an imbalanced musculotendinous development during training and maturation. We discuss evidence of non-uniform muscle and tendon adaptation in adults and adolescent athletes and the potential implications. The review concludes with proposing a concept for prevention, which targets the increase of tendon stiffness and is based on recent advancements in our understanding of tendon adaptation.

## Influential factors of imbalanced muscle-tendon adaptation and development

The following sections will briefly review the basic mechanisms of muscle and tendon adaptation and mechanotransduction. We will emphasize differences in the temporal dynamics of adaptive changes and the mechanical stimuli that effectively elicit changes of the respective tissue properties, structure and morphology. For a more comprehensive overview on muscle or tendon adaptation the reader is referred, for instance, to the reviews by Toigo and Boutellier (2006), Folland and Williams (2007), and Gonzalez et al. (2016), or Wang (2006), Magnusson et al. (2007), and Bohm et al. (2015), respectively.

### Differences in the temporal dynamics of muscle and tendon adaptation

Muscle and tendon tissue both adapt to increased mechanical loading from the subcellular to the macroscopic level. Here we give a synopsis about the changes that can be observed in the musculotendinous system that are most relevant for strength and power production. With regard to the scope of this review, we will specifically address the time course of these changes and the features of muscles and tendons that might be responsible for differences in the temporal dynamics of adaptation.

#### Basic mechanisms of muscle adaptation

The adaptive changes that affect the strength generating capacity of a muscle can be categorized into (a) radial adaptation, (b) longitudinal adaptation and (c) adaptation of specific tension (Goldspink, 1985; Bottinelli, 2001). Radial adaptation describes the modulation of the number of sarcomeres in parallel and is best reflected by changes of the physiological cross-sectional area (PCSA) of a muscle (Haxton, 1944), which is the area of the muscle cross-section perpendicular to the orientation of the fibers. In humans, an increase in muscle PCSA following strength training has been demonstrated for the elbow flexors (Kawakami et al., 1995) and knee extensors (Kawakami et al., 1995; Seynnes et al., 2009; Erskine et al., 2010). Moreover, several authors reported an increase of pennation angle after applying interventions that promote muscle strength (Aagaard et al., 2001; Blazevich et al., 2007; Seynnes et al., 2007; Farup et al., 2012). An increase of pennation angle is considered to be a modulating factor of the PCSA (Alexander and Vernon, 1975) that enables fiber hypertrophy and hence radial muscle growth to exceed the changes of the whole muscle anatomical cross-sectional area (ACSA; Häkkinen et al., 1998; Aagaard et al., 2001). The increase in single muscle fiber cross-sectional area that governs the radial muscle adaptation is the main contributor to the increasing force generating potential of the muscle (Johnson and Klueber, 1991; Aagaard et al., 2001; Farup et al., 2012) and is in turn attributed to increased myofibrillar growth (McDougall et al., 1980) and proliferation (Goldspink, 1970). Longitudinal muscle adaptation refers to the modulation of the number of sarcomeres in series, which is positively associated with the maximum shortening velocity and mechanical power of muscle fibers (Goldspink, 1985). Animal models (Lynn and Morgan, 1994; Butterfield et al., 2005) and indirect evidence from human *in vivo* studies (Blazevich et al., 2007; Duclay et al., 2009; Potier et al., 2009; Reeves et al., 2009; Franchi et al., 2014; Sharifnezhad et al., 2014) both support the notion that eccentric loading can induce longitudinal muscle plasticity. Specific tension (or force) refers to the intrinsic strength generating capacity of the muscle tissue (i.e., active force normalized to PCSA upon maximum activation). However, in light of the conflicting results on loading-induced changes of single-fiber specific tension (Widrick et al., 2002; D'Antona et al., 2006; Pansarasa et al., 2009) it is still unclear if the modulation of specific tension is a relevant contributor to strength gains in response to exercise (Folland and Williams, 2007).

#### Basic mechanisms of tendon adaptation

The early work of Ingelmark (1945, 1948) already suggested that tendons adapt to their mechanical environment. When a muscle-tendon unit is repeatedly exposed to increased mechanical loading, for instance by means of resistance exercise, it is commonly observed that the associated gains of muscle strength are accompanied by an increase of tendon stiffness (Kubo et al., 2001a; Arampatzis et al., 2007; Kongsgaard et al., 2007). When a shortening of the tendon is ruled out as a potential contributor, two candidate mechanisms can account for exercise-induced increases of tendon stiffness: (a) changes of the material properties (i.e., elastic modulus) and (b) radial tendon hypertrophy. Exercise intervention studies on human adults that reported an increase of tendon stiffness almost exclusively (with the exception of Kongsgaard et al., 2007) also found the tendon elastic modulus increasing by 17–77% (Kubo et al., 2001a; Arampatzis et al., 2007, 2010; Seynnes et al., 2009; Carroll et al., 2011; Malliaras et al., 2013b; Bohm et al., 2014). In comparison, indications of tendon hypertrophy were documented less consistently, with some evidence of moderate increases of tendon cross-sectional area (CSA; 4–10%) in response to increased mechanical loading (Arampatzis et al., 2007; Kongsgaard et al., 2007; Seynnes et al., 2009; Bohm et al., 2014), and several reports of increased tendon stiffness without concomitant radial tendon growth (Kubo et al., 2001a, 2002, 2007, 2010a; Arampatzis et al., 2010; Carroll et al., 2011; Malliaras et al., 2013b). However, cross-sectional comparisons between athletes and untrained adults suggest that tendon hypertrophy of 20–35% is well possible (Rosager et al., 2002; Magnusson and Kjaer, 2003; Kongsgaard et al., 2005; Seynnes et al., 2013). Negating potential selection bias and intersubject variations by comparing the dominant with the nondominant leg of badminton players and fencers, Couppé et al. (2008) found habitually increased loading to result in an average increase of tendon CSA of about 20%. Therefore, tendon hypertrophy is currently considered to contribute to increased tendon stiffness following long-term mechanical loading.

#### Temporal dynamics of adaptation

It is evident that both muscle and tendon are responsive to mechanical loading. However, the metabolism of tendons is designed to meet the functional demand of bearing loads over long durations and, thus, tendon tissue needs to tolerate low oxygen tension (Józsa and Kannus, 1997). Therefore, tendon tissue is characterized by a lower cell to overall dry mass ratio, vascularization, and metabolism compared to muscle tissue (Peacock, 1959; Smith, 1965; Laitinen, 1967; Ippolito et al., 1980). The half-life of tendon collagen is estimated to be almost tenfold higher compared to the muscle proteins actin and myosin (Lundholm et al., 1981; Thorpe et al., 2010). A recent study that investigated tissue renewal by means of comparing nuclear bomb ^14^C residues in forensic muscle and tendon samples provided strong support for the hypothesis of a lower rate of tissue remodeling in tendon, especially following the formation of the tendon core tissue during adolescence (Heinemeier et al., 2013). Though similar to muscle proteins, collagen synthesis increases rapidly following exercise (Miller et al., 2005), effective tissue turnover is markedly lower, leading to the suggestion that a considerable amount of the synthesized collagen molecules is not permanently incorporated in the tissue structure but broken down relatively quickly (Heinemeier et al., 2013). In accordance with this notion, it has been demonstrated in adults that changes of muscle morphology and architecture can occur as early as after 3 to 4 weeks in a heavy resistance training intervention (Seynnes et al., 2007; DeFreitas et al., 2011), while there are no reports of such rapid adaptations of tendon morphological or mechanical properties. Moreover, neuronal adaptation enables muscle strength to increase markedly even before major morphological changes occur (Folland and Williams, 2007). An increase of tendon stiffness on the other hand relies on a modulation of tissue metabolism and subsequent adaptive changes of tissue structure and tendon morphology.

Kubo and colleagues investigated the time course of muscle and tendon adaptation in two separate 3-month exercise intervention studies on the patellar (Kubo et al., 2010b) and the Achilles tendon (Kubo et al., 2012), respectively. In both studies, a marked increase of muscle strength preceded significant changes of tendon stiffness by 1–2 month and morphological (CSA) changes occurred at the muscle level only. In contrast, Urlando and Hawkins (2007) reported no significant changes of Achilles tendon strain during maximum voluntary contractions despite an increase of tendon force determined at six time-points over an 8-week strength training intervention. This finding indicates a uniform adaptation of muscle strength and tendon stiffness. However, it is interesting to note that the individual tendon strain values showed great fluctuations between the measurement sessions. The highest single values of tendon strain measured in each session, for example, ranged from 8.6 to 13.5%. This substantial variation indicates that the time course of muscle and tendon adaptation might show an individual development and that imbalances of muscle strength and tendon stiffness might have remained undetected in the study by Urlando and Hawkins (2007) due to the analysis of group mean values only.

In conclusion, both muscle and tendon are able to adapt to a change of their mechanical environment. Though information on the time course of muscle and tendon adaptation *in vivo* is still rare, the metabolic features and adaptive mechanisms differ between muscle and tendon. Muscle seems to be characterized by a greater rate of effective tissue renewal compared to tendon, and neural adaptation further increases the plasticity of the force generating capacity of the neuromuscular system. Thus, it is possible that imbalances of muscle strength and tendon stiffness can develop during a training process.

### Differences of muscle and tendon in the responsiveness to certain mechanical stimuli

Besides the potentially different time course of adaptive changes reviewed above, substantial evidence suggest that muscle and tendon feature significant differences regarding the mechanical stimuli that effectively elicit adaptive changes. This section gives a short overview about the processes of mechanotransduction and the stimuli that seem to successfully activate the respective signaling pathways for both muscle and tendon, and then closes with a comparative discussion.

#### Mechanotransduction in muscle

It is now well known that both mechanical and metabolic stress are important, separate but interacting stimuli that trigger muscle adaptation and growth (Goldberg et al., 1975; Vandenburgh and Kaufman, 1979; Rooney et al., 1994; Schott et al., 1995; Smith and Rutherford, 1995). Briefly, mechanical stress subjected to muscle tissue leads to the activation of mechanosensitive calcium channels (Kameyama and Etlinger, 1979), intracellular enzymes and second messengers (Hornberger et al., 2006) and stimulates insulin-like growth factor I (IGF-I) release from the muscle cells (Perrone et al., 1995). These events trigger a signaling cascade via autocrine and direct intracellular pathways that results in an increase of protein synthesis (Tidball, 2005; Toigo and Boutellier, 2006; Gonzalez et al., 2016). It has been demonstrated that the increase of muscle protein synthesis following an acute bout of resistance exercise exceeds the increase of protein breakdown, given a sufficient amino acid availability provided by appropriate feeding (Rennie et al., 1982; Biolo et al., 1995; Phillips et al., 1997). The positive net muscle protein balance remains elevated for several days and contributes to the remodeling of the contractile machinery and, subsequently, to hypertrophy (Kumar et al., 2009; McGlory et al., 2017 for reviews). The increase of protein synthetic capability is mediated by an increase of myonuclear number (Allen et al., 1999). Satellite cells are activated by nitric oxide efflux of stressed myofibers (Anderson, 2000) and proliferate under the regulatory influence of IGF-I (Barton-Davis et al., 1999). The proliferated satellite cells then fuse with existing myofibers as new myonuclei (Allen et al., 1999).

Metabolic stress refers to the exercise-related accumulation of metabolites (specifically lactate and hydrogen ions). The role of metabolic stress for muscle growth in response to exercise has been attributed to the associated systemic growth-related hormone and local myokine up-regulation and/or the increased fiber recruitment with muscle fatigue (Schoenfeld, 2013; Ozaki et al., 2016). Muscle hypertrophy was consequently suggested to be driven by the interaction of mechanical and metabolic stress, and that the degree of contribution depends on the exercise modality (i.e., greater mechanical stress at high intensities and greater metabolic stress at moderate intensities; Ozaki et al., 2016). It can be concluded from this assumption that, given a sufficient overall training volume, a wide range of exercise intensities effectively elicits muscle hypertrophy, which is convincingly supported by experimental evidence (Campos et al., 2002; Tanimoto and Ishii, 2006; Mitchell et al., 2012; Schoenfeld et al., 2015, 2016).

#### Mechanotransduction in tendon

In tendon tissue, the load-induced strain of the extracellular matrix is transmitted to the cytoskeleton of the embedded fibroblast via specific transmembrane proteins (Wang, 2006; Heinemeier and Kjaer, 2011). The conformational changes of these transmembrane proteins upon load application and the activation of stretch-sensitive ion channels in the cell membrane activate intracellular signaling cascades of gene and growth factor expression for the up-regulation of collagen and matrix protein synthesis (Sackin, 1995; Chiquet, 1999; Wang, 2006; Lavagnino et al., 2015). Accordingly, studies demonstrate an increased concentration of both interstitial growth factors and binding proteins (Heinemeier et al., 2003; Olesen et al., 2006; Jones et al., 2013) as well as elevated collagen synthesis (Langberg et al., 1999, 2001; Miller et al., 2005) in mechanically loaded tendon tissue. The load-induced proliferation and collagen synthesis of tendon stem cells seems to contribute to this anabolic response of tendons to mechanical loading as well (Bi et al., 2007; Zhang et al., 2010). Further, it has been demonstrated in a rat model that mechanical loading leads to an increased production of enzymes that mediate collagen cross-linking (Heinemeier et al., 2007a), which is thought to be involved in the modulation of collagen cross-link profile in humans following resistance exercise (Kongsgaard et al., 2009).

From a mechanobiological point of view, fibroblast cell deformation and fluid flow-induced shear stress are important determinants of mechanotransduction and, thus, the adaptive response of tendons (Lavagnino et al., 2008). *In vitro* testing on the effects of cyclic load-application demonstrated that high-level magnitude strains are associated with greater tenocyte cell deformation (Arnoczky et al., 2002), collagen fiber recruitment (Kastelic et al., 1980; Hansen et al., 2002), and greater inhibition of catabolic activity (Lavagnino et al., 2003; Arnoczky et al., 2004) in comparison to lower levels of cyclic strain. These results correspond well to observations from human *in vivo* exercise intervention studies. Arampatzis et al. (2007, 2010) compared the effects of two equivolume loading regimen and found significant changes of tendon stiffness and elastic modulus of the Achilles tendon only in response to the high-strain protocols [i.e., 90% isometric maximum voluntary contraction (iMVC), corresponding to 4.6% of tendon strain], while no significant changes were induced by moderate-strain training (i.e., 55% iMVC, 2.9% tendon strain). Similar results from experimental studies on the patellar tendon (Kongsgaard et al., 2007; Malliaras et al., 2013b) and two recent meta-analyses (Bohm et al., 2015; Wiesinger et al., 2015) strengthened the conclusion that high-intensity loading is a crucial stimulus for *in vivo* tendon adaptation. The muscle contraction type (i.e., isometric, concentric, eccentric) for load application does on the other hand not seem to be of particular relevance for the adaptive response (Kjaer and Heinemeier, 2014; Bohm et al., 2015).

Interestingly, against the assumptions of finite-element modeling (Lavagnino et al., 2008), the main body of experimental *in vivo* evidence suggests that the induction of high strain rates and associated increased fluid flow-related shear stress by means of plyometric exercise fails to elicit significant adaptive changes of human tendons (Kubo et al., 2007; Fouré et al., 2009, 2010; Houghton et al., 2013), even at high-intensity loading magnitudes (Bohm et al., 2014). Though it needs to be acknowledged that there are also reports of an increase of tendon stiffness in response to plyometric loading (Burgess et al., 2007; Wu et al., 2010; Hirayama et al., 2017), some of these findings might have been biased by the lack of consideration of the contribution of antagonistic activity in the calculation of tendon forces (Wu et al., 2010; Hirayama et al., 2017), which leads to an overestimation of the increase of tendon stiffness upon a training-induced reduction of antagonistic coactivation. Yet, more importantly, the few existing studies directly comparing the effects of plyometric training to low-strain-rate loading regimen consistently show lower adaptive responses in the plyometric training groups (Burgess et al., 2007; Kubo et al., 2007; Bohm et al., 2014). It has been argued that a short duration of strain-application (e.g., high-frequency load-relaxation cycles or plyometric loading) might reduce the effectiveness of mechanotransduction processes (Arampatzis et al., 2010; Bohm et al., 2014). In a systematic experimental modulation of strain duration at high strain magnitude *in vivo*, an increase by up to 3 s facilitated tendon adaptation (Arampatzis et al., 2010; Bohm et al., 2014). However, a further increase of strain duration to 12 s per cycle did not further promote adaptive effects. It seems possible that longer sustained tendon strains become less effective if the strain duration is increased at the expense of the number of loading cycles (Bohm et al., 2014).

Collectively, current *in vivo* evidence on human tendon adaptation suggests that tendons can be most effectively strengthened if loading regimen incorporate slow repetitive high-magnitude tendon strain application. High strain rate and frequency modes of loading as, for instance, plyometric exercise, do not seem to consistently stimulate tendon adaptation.

#### Comparison of muscle and tendon mechanical stimulation

**Figure 1:**
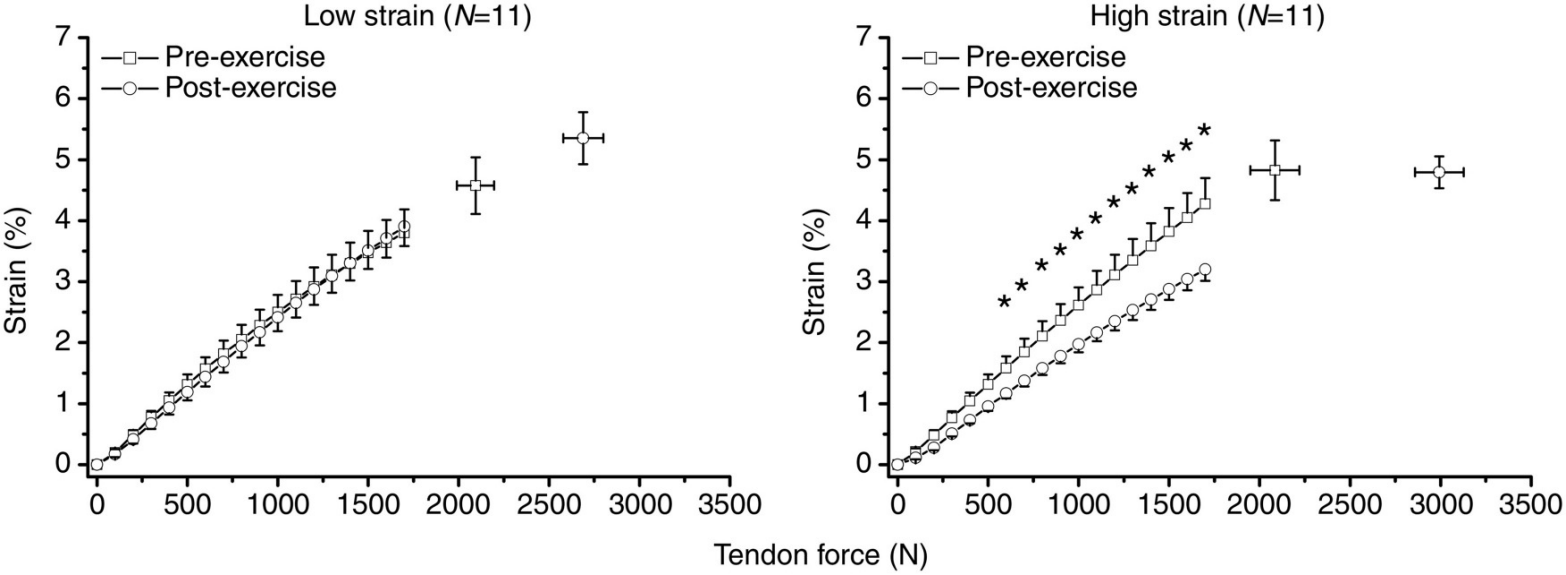
Achilles tendon-aponeurosis force-strain relationship before and after two isometric exercise protocols. The two legs of eleven adults were subjected to either moderate [i.e., 55% maximum voluntary contraction (MVC); low strain] or high loading (90% MVC; high strain) for 14 weeks, respectively. Both protocols induced an increase of tendon force. However, following moderate loading (i.e., low strain protocol) tendon stiffness did not change significantly and, thus, there was a significant increase of tendon strain during maximum muscle contractions. This implies an increase of the mechanical demand placed upon the tendon. No change was observed in a control group (data not shown). ^*^Significant difference between pre- and post-exercise values (Arampatzis et al., 2007, adapted with permission from The Company of Biologists Limited).

Comparing the types of mechanical stimulation that effectively elicit muscle compared to tendon adaptation, it seems that fatiguing training with moderate loads can trigger increases of muscle strength and size (Moss et al., 1997; Wernbom et al., 2007; Mitchell et al., 2012; Schoenfeld et al., 2016), but do not provide a sufficient stimulus for tendon adaptation (Arampatzis et al., 2007, 2010; Kongsgaard et al., 2007). This way, an increase of muscle strength without a concomitant modulation of tendon stiffness following moderate intensity loading can result in higher tendon strain during maximal voluntary contractions, which implies an increase of the mechanical demand placed upon the tendon by the working muscle (Arampatzis et al., 2007, 2010; Figure 1). Furthermore, numerous studies demonstrated that plyometric loading effectively promotes muscle strength development, also in trained athletes (Sáez-Sáez de Villarreal et al., 2010 for review), while evidence suggests that plyometric loading does not consistently increase tendon stiffness (Kubo et al., 2007; Fouré et al., 2009, 2010; Houghton et al., 2013). Accordingly, Kubo et al. (2007) reported increased tendon elongation (and, thus, tendon strain, given that the rest length did not change) during maximum muscle contractions following their plyometric training intervention. Such differences in the responsiveness of muscles and tendons to plyometric loading and its mechanical implications could be of particular significance in light of the high prevalence of tendon overuse injuries in sports with a plyometric loading profile like volleyball, basketball or athletic jump disciplines (Lian et al., 2005). Studies on growth factor transcription following loading also support the idea of differently graded responses of muscle and tendon to specific types of loading. For example, Heinemeier et al. (2007a,b) found a contraction type-specific expression of growth factors in muscle but not tendon tissue using a rat model. More recently, it was also demonstrated in humans that following a fatiguing one-leg kicking exercise with moderate loads the expression of tissue-specific growth factors increased only at the muscle but not tendon level (Heinemeier et al., 2011). This observation led the authors to conclude that an imbalanced adaptation of muscle and tendon might develop under specific loading conditions (Heinemeier et al., 2007a, 2011).

In summary, muscle responds well to a wide range of exercise modalities with an increase of strength. Tendon tissue on the other hand seems only to be responsive to high magnitude loading and repetitive loading cycles featuring long tendon strain durations show greater effects compared to modes of loading where single-cycle strain duration is short. Those differences in the responsiveness to certain stimuli might promote the development of imbalances of muscle strength and tendon stiffness in the training process in specific sport disciplines (e.g., jump disciplines).

### The effects of maturation on muscle and tendon development and plasticity

Aside from mechanical loading, maturation induces profound changes of the skeletal, neuromuscular and tendinous system in young athletes. The following section briefly reviews muscle and tendon development from child to adulthood, then focuses on somatic and hormonal changes that might challenge the balance of the development of muscle and tendon properties. The section closes with a synopsis of recent experimental evidence of an imbalanced muscle and tendon development in adolescent athletes.

#### Maturation and muscle development

Whole body muscle mass increases progressively from childhood to adulthood, with a pronounced rise during adolescence, especially in boys (Malina et al., 2004; McCarthy et al., 2014; Kim et al., 2016). Even when normalized to body mass, these changes are still marked in boys yet modest in girls (McCarthy et al., 2014; Kim et al., 2016). Studies investigating single muscle development consequently reported an increase in length, ACSA and volume (Kanehisa et al., 1995a,b; Kubo et al., 2001b; Neu et al., 2002; Tonson et al., 2008; O'Brien et al., 2010c). It seems that the gain of muscle volume is governed by both an increase in PCSA and fascicle length, yet the gains in PCSA exceed those of fascicle length in pennate muscles, which is an indication of a remodeling in favor of force production (Morse et al., 2008; O'Brien et al., 2010c; Bénard et al., 2011). Together with an increase of moment arm lengths (O'Brien et al., 2009; Waugh et al., 2012) and muscle activation (Dotan et al., 2012), this leads to a disproportionate increase of muscle strength (O'Brien et al., 2010a). The development of muscle PCSA from childhood to adulthood is most likely based on single fiber hypertrophy and not hyperplasia (Bowden and Goyer, 1960; Aherne et al., 1971; Oertel, 1988; Lexell et al., 1992). The growth hormone-IGF-I axis, which is markedly activated during adolescence for the regulation of overall body growth, stimulates fiber hypertrophy and protein synthesis (Grohmann et al., 2005). For example, myoblast proliferation and fusion with myotubes—a prerequisite for radial and longitudinal fiber growth—depends on growth hormone and IGF-I secretion (Cheek et al., 1971; Allen et al., 1999; Grohmann et al., 2005). Consequently, there is a close association between single fiber CSA and body height (Aherne et al., 1971). The alteration of the endocrine environment during adolescence, specifically the increasing systemic levels of sex steroid hormones, further initiate the development of the muscle functional, morphological and structural differences between boys and girls (Oertel, 1988; Round et al., 1999).

#### Maturation and tendon development

First information on the development of the mechanical properties of human tendinous tissue *in vivo* was provided by Kubo et al. (2001b). Their comparison of vastus lateralis tendon-aponeurosis compliance between children, adolescents and adults indicated a progressive increase of tendinous stiffness (i.e., the inverse of compliance) from childhood to adulthood, despite the longitudinal growth of the muscle-tendon unit. Theoretically, an increase in length of the series elastic elements would reduce their stiffness (given similar material properties and CSA; Butler et al., 1978). However, O'Brien et al. (2010b) found greater stiffness of the patellar tendon in adults compared to pre-pubertal children as well, and Mersmann et al. (2016) recently demonstrated an increase of patellar tendon stiffness in a longitudinal study over 1 year throughout adolescence. More detailed information on the time course of human tendon development *in vivo* is strongly limited. Kubo et al. (2014) compared the mechanical and morphological properties of the patellar tendon of elementary and high school boys to adult men. The results indicated that the major developmental increase of tendon elastic modulus from childhood to adulthood occurs until early-adolescence. This corresponds to the observations on the Achilles tendon by Waugh et al. (2012), who found that the differences in Achilles tendon material properties between younger (5–7 years) and older pre-pubertal children (aged 8–10 years) were of similar order as the differences between the latter group and the notably older adults (~26 years). Thus, the material properties of tendons might demonstrate their most pronounced development early in youth (i.e., before the growth spurt at the onset of adolescence), while it seems that tendon hypertrophy progresses further throughout adolescence (Kubo et al., 2014). This assumption also parallels observations in rodent models (Ansorge et al., 2011; Miller et al., 2012). The development can probably be attributed to a great extent to the increase of mechanical loading due to gains in body mass and muscle strength, as predicted by Waugh et al. (2012) using a stepwise multiple regression model and data of the Achilles tendon properties of children and adults. Interestingly, age was shown to be an additional significant predictor of elastic modulus in the regression model, independent of body mass and tendon stress, explaining 31 and 52% of the variance of the elastic modulus in children and both age groups combined, respectively. Thus, it seems very likely that maturation is a separate factor for tendon development, besides the effects of increased mechanical loading. Indeed, several hormones and growth factors that are involved in the regulation of somatic growth (see Murray and Clayton, 2013 for review) have been shown to mediate tendon metabolism as well. For instance, growth hormone and IGF-I stimulate gene expression, collagen synthesis and cross-linking (Abrahamsson et al., 1991; Choy et al., 2005; Doessing et al., 2010; Nielsen et al., 2014) and thyroid hormones are involved in the regulation of tenocyte proliferation, growth and collagen synthesis (Oliva et al., 2013; Berardi et al., 2014).

#### Challenges to the musculotendinous system induced by maturation

Neugebauer and Hawkins (2012) reported that the longitudinal growth of the muscle-tendon unit during adolescence can go in line with a temporary reduction of tendon CSA. At given tendon material properties, the resultant increase of tendon stress would lead to higher tendon strain. However, as Neugebauer and Hawkins (2012) found both elastic modulus and maximum tendon strain to increase only in tendency, the implications of the observed morphological development of tendons during the adolescent growth spurt still need to be elucidated. Similarly, little information is available on the effects of the rapid increase of circulating sex hormones (i.e., testosterone in boys and estrogens in girls) during puberty on the muscle-tendon unit. Yet, it seems possible that the change of the endocrine milieu could affect the uniformity of the development of muscle strength and tendon stiffness. It is well established that testosterone is one of the most potent hormones promoting muscle hypertrophy and thus increasing muscle strength (see Vingren et al., 2010 for review). Its role in the development of tendinous tissue on the other hand is basically unknown to date (Hansen and Kjaer, 2014). In adults it has been shown that anabolic-androgenic steroid supplementation stimulates collagen synthesis (Pärssinen et al., 2000) and increases tendon stiffness (Inhofe et al., 1995; Marqueti et al., 2011; Seynnes et al., 2013), but impairs tissue remodeling (Marqueti et al., 2006) and reduces ultimate stress and strain (Inhofe et al., 1995; Marqueti et al., 2011; Tsitsilonis et al., 2014). Though it seems likely that steroid supplementation is not necessarily representative of the physiological mechanisms of testosterone action, it can at least be concluded that the anabolic and strength-promoting effects of testosterone are more clearly established for muscle than tendon tissue. The effects of estrogens on muscle and tendon metabolism have been studied more extensively. In a recent review, Hansen and Kjaer (2014) concluded that current scientific evidence from studies on humans renders estrogens as muscle-anabolic, since they decrease protein turnover and increase the responsiveness to mechanical loading. Conversely, estrogens seem to reduce tendon collagen synthesis and the plasticity of tendon mechanical properties in response to exercise (Miller et al., 2007; Hansen et al., 2009). While it must be stated clearly that our current understanding of the effects of sex hormones on the muscle-tendon unit is based primarily on studies administering exogenous hormones to adults, these findings allow the hypothesis that the change of endogenous sex hormone levels in child to adulthood development could contribute to an imbalanced adaptation of muscle and tendon in youth athletes.

#### Evidence of imbalanced muscle and tendon development in adolescent athletes

**Figure 2:**
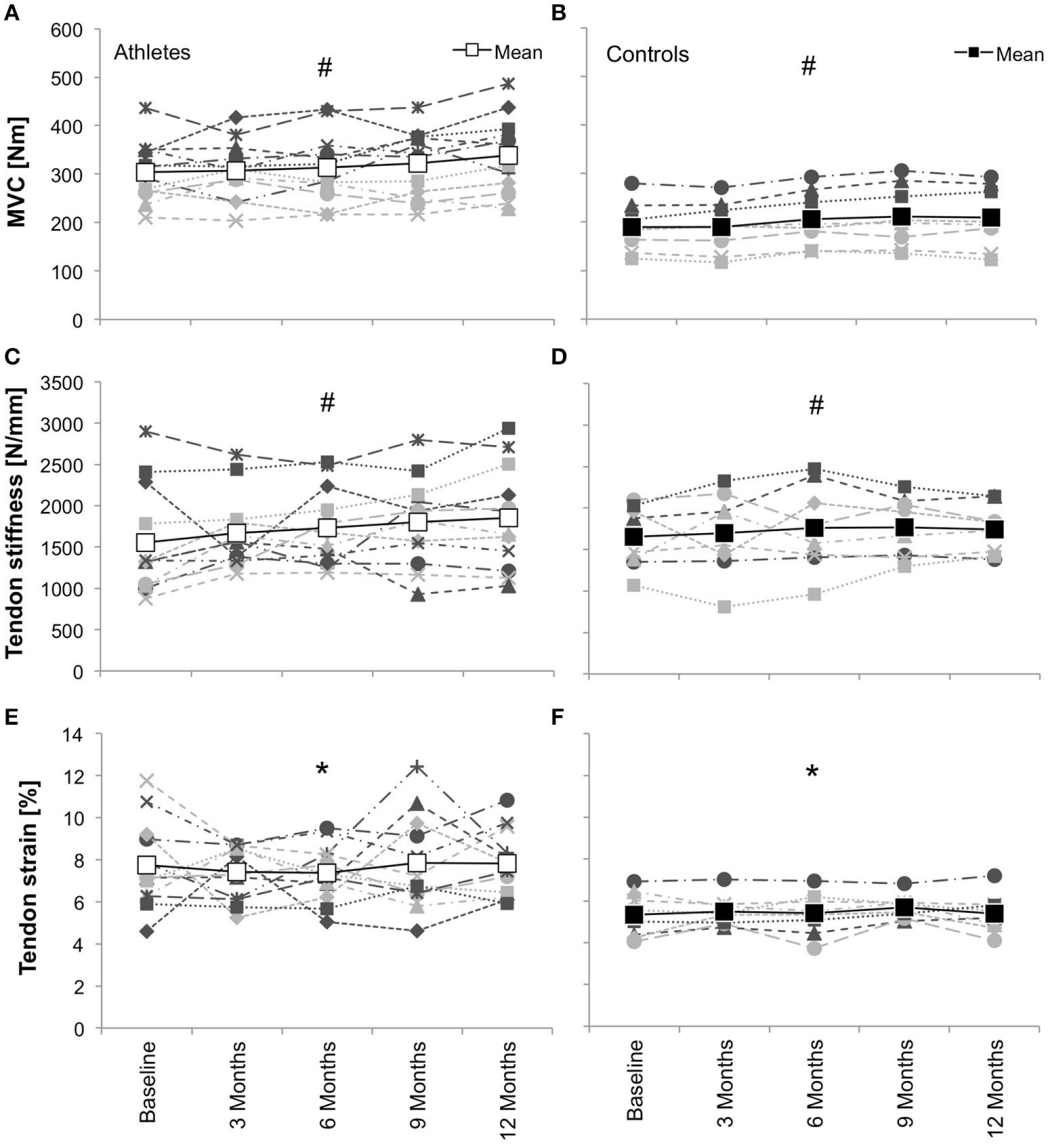
On-year development (in 3-month intervals) of knee extensor muscle strength (MVC; **A,B**), patellar tendon stiffness **(C,D)** and tendon strain during maximum contractions **(E,F)** in adolescent volleyball athletes (*n* = 12; **A,C,E**; white symbols show mean values) and controls (*n* = 8; **B,D,F**; black symbols show mean values), including individual data of female (light gray) and male participants (dark gray) in both groups. ^*^Significant difference between groups (i.e., intercept; *p* < 0.05); ^#^significant change over time (i.e., slope; *p* < 0.05). Note that differences between groups were not tested for MVC and stiffness. The data shows greater fluctuations of muscle strength in the athletes and, as a result of an imbalanced adaptation of muscle and tendon, both greater average tendon strain during maximum contractions as well as greater fluctuations of strain over time (Mersmann et al., 2016, with permission from American Physiological Society).

A first series of studies that explicitly investigated the uniformity of muscle and tendon development and adaptation during adolescence supports the idea that the two-fold stimulus of (a) maturation and (b) a predominantly plyometric loading profile can lead to a musculotendinous imbalance that increases the load (i.e., stress) and internal demand (i.e., strain) for the tendon. Mersmann et al. (2014) compared mid-adolescent to middle-aged elite volleyball athletes, which were subjected to many years of sport-specific loading. While there were no significant differences in vastus lateralis PCSA and in the force applied to the patellar tendon during maximum isometric knee extension contractions, the adolescents had a deficit with regard to patellar tendon CSA compared to the adult counterparts and, as a consequence, were subjected to increased levels of tendon stress and strain. The conclusion that the morphological plasticity of the tendon unfolds at later stages during development was supported by the results of a subsequent 2-year longitudinal study. The observations suggested that the muscular development was already far progressed in the mid-adolescent athletes, demonstrating only minor changes until the end of adolescence, while the tendon still showed remarkable radial growth and an associated increase of stiffness (Mersmann et al., 2017a). Finally, the time course of muscle and tendon development in mid-adolescent athletes was investigated in five measurement sessions over 1 year in more detail and the effects of maturation and mechanical loading were differentiated by including a similar-aged control group of non-athletes (Mersmann et al., 2016). It was found that the development in elite volleyball athletes was characterized by significantly greater fluctuations in muscle strength and a non-uniformity of muscle and tendon adaptation. Consequently, tendon strain during maximum contractions was not only increased chronically in comparison to controls, but also demonstrated significantly greater fluctuations during the period of investigation (Figure 2). In addition to the discordant changes of muscle strength and tendon stiffness that occur during a training process, results of a very recent study indicate that also the adaptive potential, at least in response to predominantly plyometric long-term loading, is lower with regard to tendon stiffness compared to muscle strength in both adolescent boys and girls (Mersmann et al., 2017b). Though from this study it still remains an assumption that maturation contributed to the development musculotendinous imbalance in addition to the unfavorable type of loading, the increased stress and strain observed in the earlier study (Mersmann et al., 2014) in adolescent athletes compared to the adults, which were habitually subjected to intense plyometric loading as well, carefully suggest that the risk might be increased during adolescence.

### Summary

Collectively, the evidence reviewed in this chapter strongly suggests that muscle and tendon show differences in the time course of adaptation to mechanical loading and in the types of mechanical stimulation that effectively elicits adaptive processes. Maturation acts as an additional stimulus on the muscle-tendon unit of young athletes and could further contribute to a development of an imbalance of muscle strength and tendon stiffness. Adolescence could be a critical phase in that context due to the associated increase of sex hormones. However, the interplay of mechanical loading and changes of the hormonal milieu on muscle and tendon plasticity in general, and with regard to adolescence in particular, is still largely unknown. Similarly, though recent evidence demonstrated that an imbalanced musculotendinous adaptation can occur during adolescence (Mersmann et al., 2014, 2016, 2017a,b), it is yet unclear how the likelihood of an imbalanced adaptation develops as a function of maturation and how it relates to specific types of loading.

## Implications and concepts for prevention

It has been described above that muscle and tendon properties might not develop homogeneously during a training process. The following chapter provides an overview about the potential implications for the risk of tendon injury based on experimental and epidemiological observations. Finally, we give recommendations for the design of preventive interventions and critically discuss the current lack of knowledge with regard to the efficacy and timing of such preventive measures.

### Potential implications for the risk of tendinopathy

#### Experimental observations

**Figure 3:**
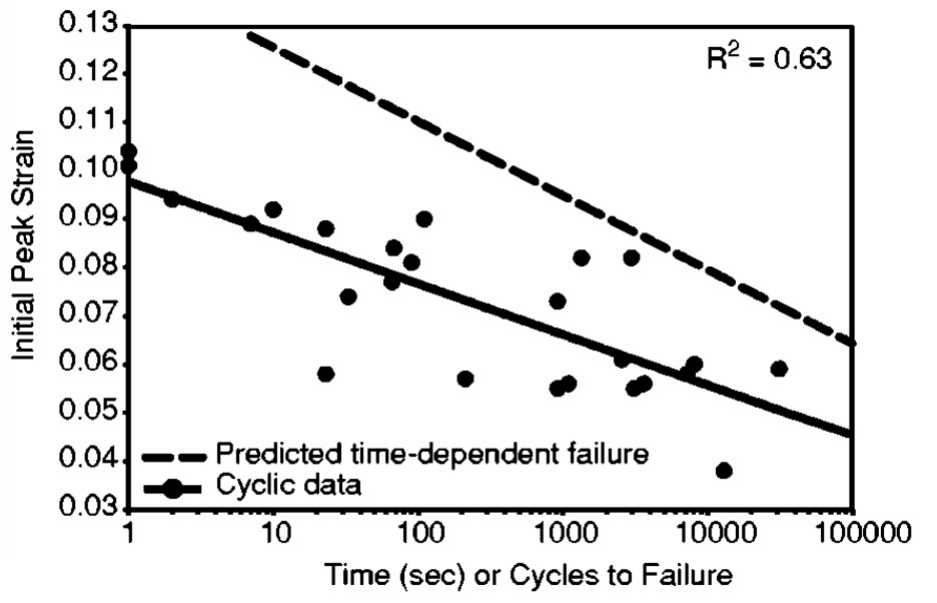
Cyclic loading lifetime results of human oder AT specimen Achilles tendons as a function of initial (peak) tendon strain during loading and associated coefficients of determination (*R*^2^). These data demonstrate that the tendon strain magnitude determines the challenge for the tissue integrity. Greater tendon strain at a given load reduces the lifetime of the tendon during cyclic loading (Wren et al., 2003, with permission from Springer).

An imbalanced adaptation of muscle and tendon—when the development of the force generating capacity of a muscle is not paralleled by an adequate change of the properties of the associated tendon—increases the mechanical demand placed upon the tendon at a given activation of the muscle and, therefore, might impose a challenge for the integrity of the tendinous tissue (especially during maximum efforts). Though tendinopathy has certainly a multifactorial etiology, the mechanical strain theory is currently considered the most probable injury mechanism and attributes the histological, molecular and functional changes of the affected tissue to mechanical overload (Archambault et al., 1995; Fredberg and Stengaard-Pedersen, 2008; Magnusson et al., 2010; Legerlotz, 2013). There is convincing evidence that repetitive loading of tendon tissue at high strain magnitudes (Butler et al., 1978; Lavagnino et al., 2006; Legerlotz et al., 2013) leads to cumulative damage in the extracellular matrix by successive collagen denaturation and fibril tears (Woo, 1982; Veres et al., 2013). The subsequent load redistribution among intact fibrils probably increases the risk of damage upon further loading cycles (Neviaser et al., 2012) and might explain the associated decrease of stiffness and ultimate stress (Schechtman and Bader, 2002; Fung et al., 2009, 2010; Legerlotz et al., 2013). The manifestation of tendinopathy is commonly ascribed to the progression of cumulative microtrauma to higher structural levels of the tissue and successive matrix breakdown (Kannus, 1997; Cook and Purdam, 2009). The degenerative cascade might also be related to the discontinued mechanotransduction of ruptured fibrils (Knörzer et al., 1986) and the associated catabolic responses of under-stimulated fibroblasts (Arnoczky et al., 2007). As both scenarios (i.e., mechanical over- or understimulation) are based on initial strain-induced damage, it seems plausible that an imbalanced adaptation of muscle and tendon could increase the risk of overload-induced tendinopathy. Wren et al. (2003) performed static and cyclic loading experiments on human Achilles tendons and demonstrated that the initial level of strain induced by a given load was inversely correlated with time or loading cycles until failure (Figure 3). This suggests that if tendon strain increases during muscle contractions due to an imbalanced musculotendinous adaptation, repetitive loading could induce significant sub-rupture fatigue and trigger pathological processes. Indeed, there are *in vivo* studies on patients and athletes with tendinopathy, which report increased levels of tendon strain during maximum voluntary contractions (Arya and Kulig, 2010; Child et al., 2010). Though other studies did not identify increased levels of strain—which might be a methodological issue as pain reduces the level of muscle activation (Hart et al., 2010; Palmieri-Smith et al., 2013) and, thus, the tendon strain measured during voluntary contraction—they found other indications of a mechanical weakening of the tendon, like a decrease of stiffness (Helland et al., 2013) or increase of tendon stress (Couppé et al., 2013).

#### Epidemiological observations

The assumption that an imbalanced adaptation of the muscle-tendon unit could contribute to the development of overuse injuries with increasing risk during adolescence also finds support from several epidemiological observations. First, it is interesting to note that the probability of non-contact soft-tissue injury rises when training loads are increased rapidly (Gabbett, 2016). Though this increase clearly could also have different origins, it still indicates that the differing time course of muscle and tendon adaption (i.e., delayed tendon adaptation compared to the increase of muscle strength) might potentially be of clinical relevance. Second, the prevalence of tendinopathy in both elite and recreational athletes is particularly high in sports with predominantly plyometric loading (Lian et al., 2005; Zwerver et al., 2011). In adolescent volleyball players, it was found that jumping ability and the weekly hours of volleyball training (and not strength training) increase the risk of tendinopathy (Visnes et al., 2013). It seems that especially the frequency of jumps during training and competition could be a major determinant of the risk of tendon overload (Bahr and Bahr, 2014). These observations could well be related to the different responsiveness of muscle and tendon to plyometric loading and increased risk of tendon fatigue damage upon repetitive loading with high magnitude strains. Third, a recent epidemiological meta-analysis on tendinopathy in children and adolescents regularly participating in sports indicated an increasing risk with age (Simpson et al., 2016), which corresponds to the incidence of general soft-tissue overuse injuries reported earlier (Stracciolini et al., 2014). These findings underline the potential influence of maturation on the balance of muscle and tendon adaptation and the necessity to deepen our understanding of the interaction of maturation and loading.

#### Summary

In summary, the pathogenesis and epidemiology of tendinopathy provides a solid theoretical background for the hypothesis that an imbalanced adaptation of muscle and tendon could have consequences for the risk of tendon injury as (a) the resultant increased mechanical demand is a candidate mechanism to induce overload, (b) the prevalence of soft-tissue overuse injuries is high at time-points in the training process (i.e., sudden increase of loading) and in sport disciplines that favor the development of a muscle-tendon imbalance from a mechanobiological point of view, and (c) maturation seems to be a potential risk factor for the development of both tendinopathy and musculotendinous imbalances in young athletes. There certainly is a need to provide more direct support for an association between imbalanced muscle and tendon adaptation and overuse. Yet, it appears that the interaction of maturation with mechanical loading could potentially increase the likelihood of the occurrence of such imbalances, which in turn might be related to the increasing risk of tendon overuse in adolescence. However, these assumptions need to be supported by further research.

### Concepts for prevention

Following the hypothesis that an imbalanced development of muscle strength and tendon stiffness could increase the risk for tendon overuse injury, it might be promising to target the increase of tendon stiffness in groups at risk (e.g., athletes of jump disciplines, adolescents commencing with resistance exercise). The following chapter provides evidence-based suggestions for an effective tendon training and comments on open issues. The chapter concludes with a critical discussion of the current evidence on the effects of preventive interventions outlines the anticipated effects of an intervention-induced increase of tendon stiffness on athletic performance.

#### Effective tendon training

**Figure 4:**
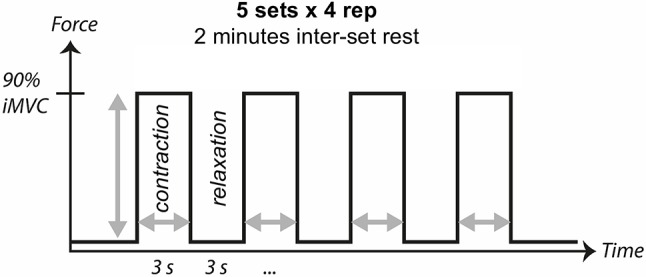
Evidence-based recommendations for an effective stimulus for tendon adaptation. High intensity loading to the tendon (85–90% iMVC) should be applied in five sets of four repetitions with a contraction and relaxation duration of 3 s each, and an inter-set rest of 2 min. We suggest the training to be applied three times a week for at least 12 weeks.

Current evidence on human tendon adaptation *in vivo* (see section Mechanotransduction in Tendon) suggests that both contraction intensity and contraction duration need to exceed a certain threshold to provide an efficient training stimulus. The training intensity is considered to be optimal around 85–90% of iMVC and the contraction duration around 3 s (Arampatzis et al., 2010; Bohm et al., 2014, 2015; Wiesinger et al., 2015). The contraction mode (i.e., isometric, concentric, eccentric) does not seem to be relevant (Kjaer and Heinemeier, 2014; Bohm et al., 2015), however, it needs to be considered that during classic dynamic training (i.e., eccentric-concentric exercises) the necessary high tendon forces occur only in specific ranges of joint angles due to the change of gear ratios during movement (e.g., between 60° and 100° of knee flexion in a parallel squat; 0° = full extension) (Flanagan et al., 2003; Peñailillo et al., 2015). Therefore, it is recommendable to increase the movement duration (e.g., to ~6 s) when a large range of motion is used during the exercise. Isometric training should be performed in joint angles close to the optimum for force generation (i.e., ~60° knee flexion for patellar tendon training or ~10° ankle dorsiflexion and extended knee for the training the Achilles tendon) and has the advantage that the training stimulus in terms of intensity and duration can be controlled quite easily. Moreover, it does not require the complex technical skills of free weight training for a safe execution and can be performed without expensive equipment (e.g., using non-elastic slings). Figure 4 illustrates a training stimulus for increasing tendon stiffness based on the most effective training protocol of a series investigations that systematically modulated mechanical strain parameters (Arampatzis et al., 2007, 2010; Bohm et al., 2014). We suggest to apply the training protocol three times a week for at least 12 weeks. These recommendations also correspond to the conclusions of two recent meta-analyses on tendon adaptation (Bohm et al., 2015; Wiesinger et al., 2015). Suggestions for specific exercises are provided as Supplementary Material to this review.

There are several important, but currently open issues that need to be elucidated with regard to the stimulation of tendon adaptation in general, its implementation in elite athletic training as a preventive measure in particular and with regard to its application in youth sports. There have been experimental investigations systematically modulating strain magnitude, rate, duration and frequency (Arampatzis et al., 2007, 2010; Kongsgaard et al., 2007; Bohm et al., 2014), however, the effects of overall training volume, number of sessions per week or rest between sets are basically unexplored thus far, and meta-analyses are limited in their potential to improve our understanding in this regard (Gentil et al., 2017) due to the heterogeneity of intervention studies (Bohm et al., 2015). In a recent study, Waugh et al. (2017) modulated inter-cycle rest duration (i.e., 3 and 10 s) between two equivolume isometric loading protocols. While the improvements of stiffness and material properties were similar between protocols, some potentially unfavorable structural changes were detected by means of ultrasound tissue characterization (UTC) following the short rest loading. It should be noted that it is still unclear if such changes of UTC-parameters indicate overload and the overall loading volume in the study by Waugh and colleagues was 2.5 times the volume of the program that we recommend based on our experience. Nevertheless, these findings highlight the necessity to deepen our understanding of tendon tissue recovery during cyclic loading, especially with regard to injury prevention and rehabilitation.

It is currently a matter of speculation when to implement the preventive intervention during the course of athletic training, if the training stimulus should be applied continuously or in periods, and how a specific tendon training interferes with training regimen that target other determinants of athletic performance. Given the moderate overall loading volume of the program recommended in this review, it seems realistic to implement the tendon exercises without any major reductions of other training contents. It can be considered that the program increases muscle strength as well (Arampatzis et al., 2007, 2010) and, therefore, could complement or replace some routines used for muscle strength development. We are currently applying a preventive tendon training in adolescent athletes to investigate its efficacy. The overall duration of exercises implemented in the regular training schedule that target the increase of tendon stiffness is around 15 min. Considering this low time-effort and the uncertainty if training at specific time-points during a season would be equally effective, we are applying this intervention over the whole season. Future research might help to further develop this approach and investigate if scheduling tendon training in advance of marked increases of loading that is assumed to provide a more potent stimulus for muscle strength compared to tendon stiffness development (i.e., plyometric loading or fatiguing moderate intensity loading) yields similar results.

The recommendations given in this chapter are based on our current knowledge on tendon adaptation, which is derived from studies on adults. Though it is known that the tendons of pre-pubertal children and adolescents are able to adapt to mechanical loading (Waugh et al., 2014; Mersmann et al., 2017b), dose-response relationships specific for the immature tendinous system still need to be established in the future. However, as we are convinced that the basic mechanisms of mechanotransduction should not be profoundly different in children and adults, we can argue that it is likely that the characteristics of an effective stimulus for tendon adaptation (i.e., high strain magnitude and ~3 s strain duration applied with low frequency) is widely independent of age. Nevertheless, there might be restrictions with regard to overall mechanical loading and intensity control. The number of sets and repetitions could then be used to adjust the overall training volume to the loading capacity of the young athletes. Moreover, in age groups where maximum strength testing is contraindicated, perceived exertion scales can be used to estimate training intensity (Waugh et al., 2014).

In conclusion, adult tendons, and likely immature tendons as well, receive effective mechanical stimulation upon high strain magnitude loading with an appropriate strain duration. Though the role of recovery is widely unexplored, the recommendations outlined above could have the potential to decrease the likelihood of an imbalanced muscle and tendon development in a training process. However, it needs to be stated clearly that specific considerations for the implementation in athletic training schedules of different sports and for the application in youth athletes need to be elucidated in future research.

#### Potential effects on risk of injury and athletic performance

The prevention or reduction of musculotendinous imbalances could, in our view, have beneficial effects on (a) the risk of tendon injury and (b) athletic performance. A recent systematic review on the effects of preventive interventions for tendinopathy concluded that evidence for their efficacy is only limited (Peters et al., 2016). However, the exercise interventions examined were either not targeting the improvement of the mechanical properties of the tendon (e.g., balance training, stretching) or did not apply training stimuli that are in accordance with the current view on the mechanobiological basis of human tendon adaptation *in vivo* (e.g., Alfredson eccentric training or Silbernagel's combined concentric-eccentric exercise; Malliaras et al., 2013a for a discussion). Conventional eccentric training approaches, for example, are characterized by high training volume but only moderate load intensity (i.e., body weight), which might even increase discrepancies between muscle strength and tendon stiffness (Arampatzis et al., 2007, 2010). Though conventional eccentric training is associated with pain relief in patients with tendinopathy, its application as a preventive measure can also increase injury risk of tendons that already feature structural abnormalities (Fredberg et al., 2008). A load-based intervention with scientific evidence supporting its efficacy on promoting tendon stiffness is likely to be a more effective preventive strategy following the hypothesis that an imbalance of muscle and tendon predispose for tendinopathy. An increase of stiffness that parallels the increase of the force generating capacity of the neuromuscular system would serve as a protective mechanism against increased strain during maximum muscle contractions. Moreover, if the increase of stiffness is governed by radial hypertrophy upon long-term training, this would also reduce tendon stress.

Aside from the potentially beneficial effects for the health of young athletes, tendon stiffness and the interaction of muscle and tendon during movement are important contributors to movement performance. For example, increased tendon stiffness is associated with a lower electromechanical delay, a greater rate of force development and jump height (Bojsen-Møller et al., 2005; Waugh et al., 2013). Certainly, the elasticity of the tendon and the associated storage and release of mechanical strain energy are important contributors to sportive performance as well (Roberts, 1997; Kawakami et al., 2002). Greater elongation at a given force allows more energy to be stored in the tendon and has been positively associated, for example, with sprint performance (Stafilidis and Arampatzis, 2007; Kubo et al., 2011). However, a greater muscle force output combined with higher tendon stiffness increases the potential exchange of mechanical energy between muscle and tendon as well (Wu et al., 2010). Moreover, if the force production during a movement task increases due to an increase of the muscular capacity, an increase of stiffness of the series elastic elements is necessary to maintain fascicle kinetics within an optimal working range (Lichtwark and Wilson, 2007). For example, it has been recently shown that vastus lateralis fascicles operate around optimum length and close to optimum velocity for power production (Nikolaidou et al., 2017) during vertical jumping. A change of the balance of muscle strength and series elastic stiffness could lead to a distortion of the musculotendinous interaction and in turn increase the demand for neuromuscular control (i.e., due to a change of fascicle kinetics), which could reduce the extent to which the increased muscular capacity can be exploited. Therefore, it is likely that a facilitation of tendon stiffness in line with muscle strength would lead to greater improvements of movement performance as opposed to a sole increase of muscle strength.

## Conclusions and future directions

Current scientific evidence strongly supports the idea that the development of muscle strength during a training process is not necessarily accompanied by an adequate modulation of tendon stiffness. The differences in the time course of adaptation and in the mechanical stimuli that trigger adaptive processes provide two mechanisms that can account for a dissociation of the muscular and tendinous development. Though the additional influence of maturation is still a heavily under-investigated topic, it is likely that an imbalanced development of muscle strength and tendon stiffness is a relevant issue for youth sports and it seems that the risk might even be increased compared to adults. Adolescence, with its associated somatic and endocrine processes, could be a critical phase in that regard. Due to the mechanical loading profile, musculotendinous imbalances especially concern athletes from jump disciplines and the high prevalence of tendinopathy in those sports as well as the increasing incidence during adolescence support the hypothesis that imbalances of muscle strength and tendon stiffness could have implications for the health of young athletes. The implementation of interventions targeting the improvement of tendon mechanical properties could be a promising approach to prevent such imbalances, promote athletic performance and reduce the risk of tendon injury. However, there is still a clear lack of information on the time course of changes of the musculotendinous system during premature development and the interaction of maturation and mechanical loading. The effects of changing sex hormone levels on tendon properties and plasticity is also widely unknown. Similarly, the association of musculotendinous imbalances with tendon overuse injury as well as the preventive value of interventions that promote the development of tendon mechanical properties has not been established thus far. The effects of recovery for tendon adaptation in general are largely unexplored and a future challenge with regard to the application of preventive tendon training in youth sports is the determination of age-specific dose-response relationships and the implementation in the training schedule in elite sports. The increasing prevalence of tendinopathy in athletic adolescents certainly calls for further research on these issues.

## Author contributions

All authors substantially contributed to the interpretation of the literature addressed in this review. FM drafted and finalized the manuscript. SB and AA made important intellectual contributions in revision of all sections of the manuscript. AA supervised the preparation of the manuscript. All authors approved the final version of the manuscript and agree to be accountable for the content of the work.

### Conflict of interest statement

The authors declare that the research was conducted in the absence of any commercial or financial relationships that could be construed as a potential conflict of interest.
