# Supplementary material for: Imbalances in the Development of Muscle and Tendon as Risk Factor for Tendinopathies in Youth Athletes: A Review of Current Evidence and Concepts of Prevention
Source: Front Physiol. 2017 Dec 1;8:987. doi: 10.3389/fphys.2017.00987 (PMC5717808; doi:10.3389/fphys.2017.00987)
Supplement: Supplementary file 1 [file Presentation1.PDF]

frontiers

Supplementary Material

Imbalances in the development of muscle and tendon as an issue for youth athletes: A review of current evidence and concepts of prevention

Falk Mersmann[1,2], Sebastian Bohm[1,2] & Adamantios Arampatzis[1,2*]

*** Correspondence:** Adamantios Arampatzis: a.arampatzis@hu-berlin.de

1 Supplementary Figures

See below.

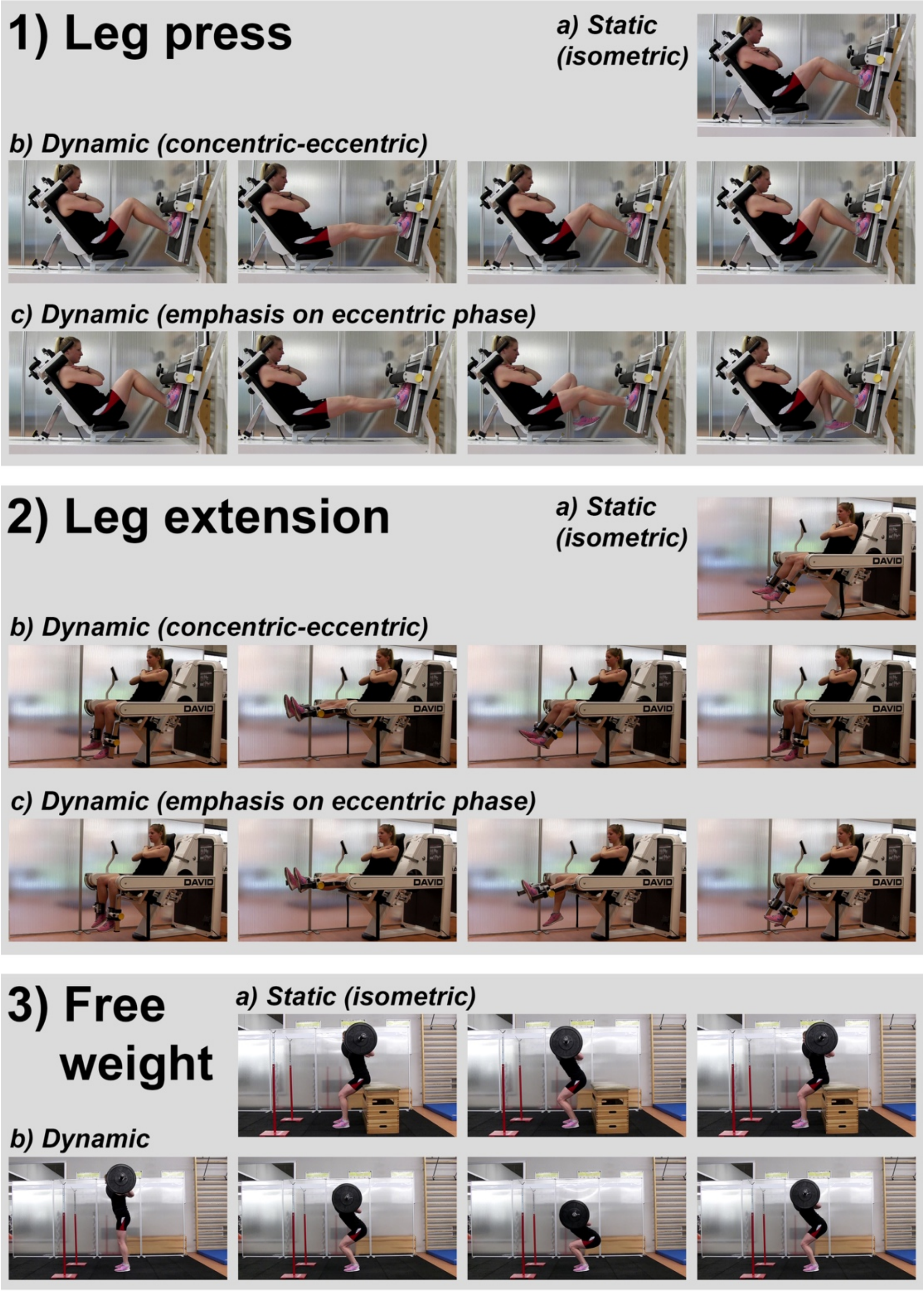

Supplementary Figure 1. Exemplary exercises for strengthening the patellar tendon on a leg press machine (1), leg extension machine (2) or using free weights (3). Repetitive isometric knee extensions at high contraction intensities (≥ 85% of voluntary maximum; 3 s contraction duration) and ~70° knee joint angle provide an efficient and easily controllable mode of loading (a). Alternatives are dynamic exercises as classical concentric-eccentric series (1b, 2b), squats (3b) or series with an emphasis on the eccentric phase (both legs lift the load in the concentric phase, yet only one bears the load during the eccentric part; 1c, 2c). The dynamic exercises should be performed slowly to ensure an adequate loading duration in the joint angle range where the necessary high tendon forces occur (i.e., we recommend an overall movement duration of 6 s to achieve a high-magnitude strain duration of ~3 s).

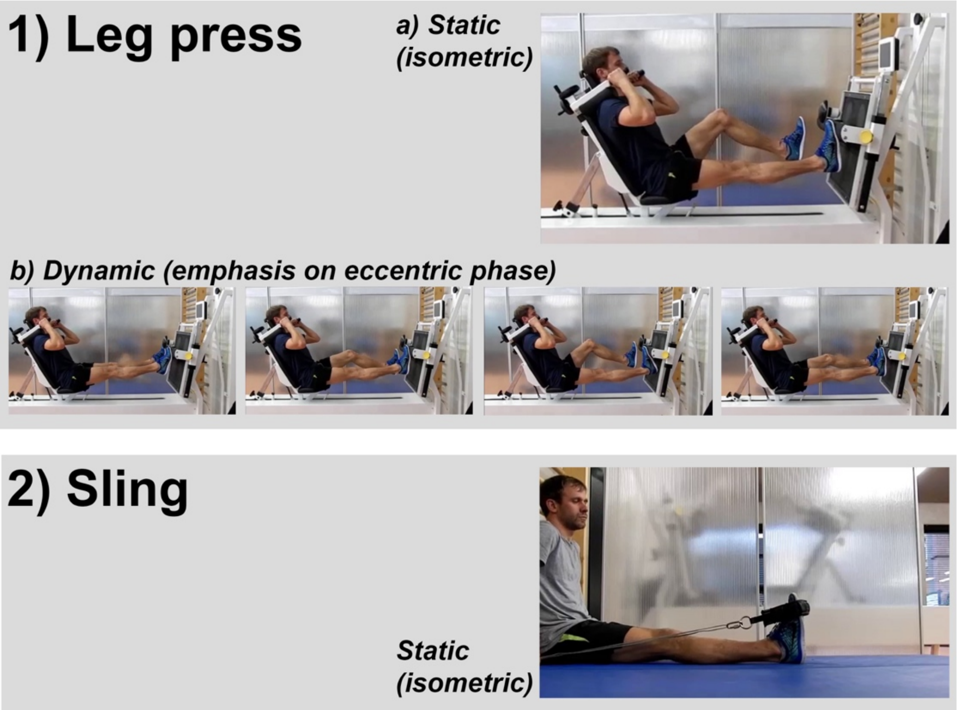

Supplementary Figure 2. Exemplary exercises for strengthening the Achilles tendon. Repetitive isometric plantar flexions at high contraction intensities (≥ 85% of voluntary maximum; 3 s contraction duration) in a neutral ankle joint position (and extended knee) provide an efficient and easily controllable mode of loading that can be performed, for example, on strength machines (1a) or using non-elastic strings (2). Alternatives are dynamic exercises as classical concentric-eccentric series or with an emphasis on the eccentric phase (both legs lift the load in the concentric phase, yet only one bear the load during the eccentric part; 1b). The dynamic exercises should be performed slowly to ensure an adequate loading duration in the joint angle range where the necessary high tendon forces occur (i.e., we recommend an overall movement duration of 6 s to achieve a high-magnitude strain duration of ~3 s).
